# Knowledge and perceptions of brucellosis in the pastoral communities adjacent to Lake Mburo National Park, Uganda

**DOI:** 10.1186/1471-2458-14-242

**Published:** 2014-03-10

**Authors:** Catherine Kansiime, Anthony Mugisha, Fredrick Makumbi, Samuel Mugisha, Innocent B Rwego, Joseph Sempa, Suzanne N Kiwanuka, Benon B Asiimwe, Elizeus Rutebemberwa

**Affiliations:** 1 Department of Health Policy Planning and Management, Makerere University School of Public Health, College of Health Sciences, P. O Box 7072, Kampala, Uganda; 2 Department of Veterinary Medicine, College of Veterinary Medicine, Animal Resources and Bio-security, P. O Box 7062, Kampala, Uganda; 3 Department of Epidemiology and Biostatistics, Makerere University School of Public Health, College of Health Sciences, P. O Box 7072, Kampala, Uganda; 4 Department of Biological Sciences, College of Natural Sciences, Makerere University, P. O Box 7062, Kampala, Uganda; 5 Infectious Disease Institute, Mulago Hospital Complex, Kampala, Uganda; 6 Department of Medical Microbiology, College of Health Sciences, Makerere University, P. O Box 7072, Kampala, Uganda; 7 Ecosystem Health Initiative, College of Veterinary Medicine, University of Minnesota, St. Paul, MN, USA

## Abstract

**Background:**

Brucellosis is one of the most common zoonotic infections globally. Lack of knowledge about brucellosis may affect the health-seeking behavior of patients, thus leading to sustained transmission in these communities. Our study assessed knowledge and perceptions of brucellosis among pastoral communities adjacent to Lake Mburo National Park (LMNP), Kiruhura District, Uganda.

**Methods:**

A community cross-sectional questionnaire survey involving 371 randomly selected household heads from three sub-counties neighboring LMNP were interviewed between June and August 2012. Data collected included communities’ knowledge on causes, symptoms, transmission, treatment, prevention and risk factors of brucellosis. Multivariable logistic regression analysis was performed to explore strength of association between overall knowledge of brucellosis and various individual factors using odds ratios and 95% confidence intervals.

**Results:**

Only 70 (19%) knew the symptoms of brucellosis in animals, and three quarters (279, 75.5%) mentioned joint and muscle pain as a common symptom in humans. Almost all participants (370, 99.3%) had ever heard about brucellosis, majority (311, 84.7%) believed it affects all sexes and two thirds (67.7%) of the respondents believed close proximity to wildlife contributes to the presence of the disease. Almost all (352, 95.4%) knew that brucellosis in humans could be treatable using modern drugs. The main routes of infection in humans such as consumption of unpasteurized dairy products were known by 97% (360/371); eating of half-cooked meat by 91.4% and eating contaminated pasture in animals by 97.4%. There was moderate overall knowledge of brucellosis 197 (53.1%). Factors associated with higher overall knowledge were being agro-pastoralists (aOR: 2.08, CI: 1.17-3.71) compared to pure pastoralists while those who reported that the disease was a health problem (aOR: 0.18, CI: 0.06-0.56) compared to those who said it was not were less likely to be knowledgeable.

**Conclusions:**

There was moderate overall knowledge of human and animal brucellosis among the participants. Majority of the participants believed that close proximity to wildlife contributes to the presence of the disease in the area. There is a need for collaboration between the public health, veterinary and wildlife sectors to provide health education on brucellosis for better management of the disease in the communities.

## Background

Brucellosis is one of the most common zoonotic infections globally [1], transmitted to humans through consumption of unpasteurized dairy products or through direct contact with infected animals, placentas or aborted foetuses. Clinically, the disease is characterized by fever, fatigue, headache, sweating, joint pain, loss of appetite, muscular pain, lumbar pain, weight loss, and arthritis [2]. In humans, brucellosis is often easily misdiagnosed as other febrile syndromes such as malaria and typhoid fever, thereby resulting in mistreatments and underreporting [3].

This bacterial disease is a zoonosis of veterinary, public health and economic significance in most developing countries [4]. In livestock, it results in abortion, reduced fertility, weak offspring and lowered milk production [5]. Furthermore, the disease has major economic consequences due to time lost by patients from normal daily activities [6] with concomitant loss of income [7] and losses in animal production [8]. Therefore, in regions where human brucellosis is endemic, there is a great need to link animal and human health sectors since veterinary and public health sectors share the common goal of protecting, promoting and improving the health and wellbeing of human populations [9].

In endemic countries, such infection in humans is either via unpasteurized milk products or by exposure to infected placental material, aborted foetuses or infected animals, which after abortion can shed a vast amount of bacteria [10]. The use of dried dung as fuel and insulation in houses may also promote infection in households [6]. Brucellosis is an occupational disease; farmers, veterinarians, and inseminators are at higher risk of contracting it. There is an even stronger association with poverty; poor people live closer to their animals, are more likely to consume unpasteurized milk products and meat from infected animals, and are less prone to protect themselves when dealing with foetal fluids and vaginal discharges after abortion or full-term parturition. Furthermore, as with other conditions; poor people, especially in rural areas, are less likely to get proper diagnosis and treatment, and since brucellosis is a zoonosis it is a double burden for it affects both people and their animals - in poor households [5].

In Uganda, human Brucellosis has been reported to be prevalent in both rural and urban areas [11] with a recent case–control study at Mulago national referral hospital in Kampala showing that living in a slum area was a risk factor for infection for urban dwellers. A study of brucellosis in cattle in Mbarara district reported a herd prevalence of 55.6% and animal prevalence of 15.8% in the pastoral dairy system [12] but higher figures of up to 100% at herd level and 30% at animal level have been reported in the Central district of Nakasongola [13]. In rural Uganda, pastoralist communities are particularly at risk of the disease because of their itinerant lifestyle and other cherished ways of life that expose them to contact with animals and their products.

Since the 1960s, the government of Uganda has actively encouraged its traditionally mobile pastoralists to be sedentary. As a result, most of the former nomadic pastoralists who occupy Uganda’s cattle corridor, are now sedentary in a geographic space increasingly coming under stress from other competing land use types and climate change [14]. Among these are pastoralists in Kiruhura district, Southwest Uganda, who have been resettled and some of them living close to Lake Mburo National Park. This group has access to the park during dry seasons for pasture and water. However, this interaction between humans, domestic and wild animals is a potential threat since wildlife brucellosis also represents a potential zoonotic threat [15] as a result of spillover of infection from wildlife to cattle and later to humans in these areas. Lack of sufficient knowledge of the disease, the absence of effective prevention and management strategies and continuous interaction of human, domestic animal and wildlife may contribute to continuous spread of the disease. The aim of this study was to assess knowledge and perceptions about causes, symptoms, mode of transmission, prevention and treatment of brucellosis among pastoral communities adjacent to LMNP, Southwest Uganda. The findings of this study may contribute to the designing of control strategies of the disease in the communities.

## Methods

### Study area and population

The study was conducted in the pastoralist rangelands of Lake Mburo National Park (LMNP), Nyabushozi County, Kiruhura district, Western Uganda. The district is characterized by typical semi-arid savannah grasslands with an estimated population of 212,087 with an annual growth rate of 3% [16]. Three sub-counties of Nyabushozi were purposively chosen on the criterion of being adjacent to LMNP. The study sub-counties were Kanyaryeru to the West, Nyakashashara to the East, and Sanga to the North because of the increasing wildlife-human-domestic animal interface, hence potential risk for zoonoses [17]. Currently, there are no communal lands in this area except for cattle passages and communal watering dams adjacent to LMNP. The communities in this region derive their livelihoods by selling cattle, cattle products and recently have started growing food crops at a subsistence level. In this study we focused on three categories of pastoralists: the farmers who are settled and solely grow crops, the agro-pastoralists who rear cattle as well as practice farming and the pure pastoralists/semi-nomads who only rear cattle and these have permanent shelters but move their animals in dry seasons in search of water and pasture. The majority of cattle keepers in this area are agro-pastoralists.

### Study design and sampling procedure

Between June and August 2012, a community-based cross sectional survey was conducted in randomly selected households of three sub-counties in Kiruhura district to assess the knowledge and perception of the communities about brucellosis. With the help of the Local Council Chairmen, the research obtained a list of households from the village registers in the purposively selected sub-counties adjacent to LMNP. Villages close to the park had a minimum of 60 households. A total of ten [10] villages were purposively selected (those adjacent to the park), and from each 120 households were randomly selected. A systematic random sampling method, with a sampling interval of 5 obtained by dividing the total number of households in each village by the desired number of households; 60/12 was used to select the participating households. Study eligibility was based on willingness to be interviewed and being a household head or spouse or a person in-charge of the household aged eighteen and above in the absence of the household head and the spouse. Sample size was estimated at 354 participants from all the three sub-counties using 83.2% proportion of knowledge of brucellosis [18], a 5% level of precision of the estimate, a non-response rate of 10% and a design effect of 1.5 because participants were being selected from three independent sub-counties using multi-stage sampling. However, a total of 371 participants were enrolled in the study.

### Study design and data collection

Information on knowledge about cause, symptoms, mode of transmission, prevention, treatment and risk factors for brucellosis was collected using structured interviewer administered questionnaires. The questionnaire was translated from the original English version into the local language (Runyankole) and back translated to English by independent persons to ensure consistency, clarity and socio-cultural acceptability in the communities. During pre-testing, additional information was gathered and some of the questions were modified. The participants were interviewed in their local language by the principal investigator and trained research assistants selected from the localities. Information on the socio-demographic characteristics of the participants was also included in the questionnaires. Upon completion of the questionnaire, the interviewers provided household members with relevant disease information and gave the participants the opportunity to ask questions about brucellosis. Disease information in both animals and humans included a description of brucellosis and its cause, symptoms, the potential routes of transmission, treatment, and measures to prevent infection.

### Data analysis

Collected data were coded and entered into a data base using Epi-Data software version 3.1, while analysis was conducted using STATA soft-ware Version 10. Categorical variables were presented as proportions and their associations determined by Chi-square test. Bivariate analysis was performed to explore associations between overall knowledge and independent variables such as age, sex, marital status, education, occupation, family ever had member with brucellosis, ever heard about brucellosis, whether it was a health problem in the area and if it was preventable. Furthermore, bivariate analysis was done for subscale domain of knowledge (symptoms, transmission routes, treatment, and preventive methods in both humans and animals) and risk factors. Associations were considered to be statistically significant if they achieved a p < 0.05. Logistic regression models were fitted to estimate independent associations between subscale knowledge and predictor variables. Variables for sub-scale knowledge were collected as multiple responses, and for one to be considered knowledgeable about brucellosis; he or she would have given at least two or more correct responses for each category of knowledge.

Inclusion of variables into the multivariable analysis was based on factors in bivariate analyses that either had p ≤ 0.2 or known potential cofounders or factors associated with knowledge from previous studies. Overall knowledge (dependent variable) of human and animal brucellosis was assessed on correct knowledge of symptoms (in animals and humans), mode of transmission (domestic animals to humans, domestic animal to domestic animal, wild animals to domestic animal, wild animals to humans and vice versa), effective treatment (in animals and humans), and preventive methods (animal to humans and animal to animal) and risk factors. Responses to these questions were collected and analyzed as multiple responses, but a composite knowledge score generated ranging 0–16. Normal distribution of these scores was assessed by histogram. Basing on percentiles, overall knowledge of brucellosis was categorized into moderate and high knowledge using the 50^th^ percentile as cut off and 75^th^ percentile respectively. A backward stepwise method was used in order to identify the variables that were removed from the model. The least significant variables were considered first for removal. Any variables that caused an insignificant increase in deviance on removal from the model were left out of the model while the variable that caused a significant increase in deviance on removal was retained in the model. Variables removed from the model when a backward stepwise method was performed and those known to be potential cofounders or factors associated with knowledge from previous studies were tested for confounding, any of the mentioned variables that had a more than ten percentage change (>10%) in the crude and adjusted odds ratio was considered a confounder. However, we found no confounding. A goodness-of-fit test using Hosmer-Lemeshow test was conducted and found that the final model was good (*P* = 0.91).

### Ethical issues

The study protocol was approval by Makerere University School of Public Health Higher Degrees, Research and Ethics Committee as well as Uganda National Council for Science and Technology. The study objective was explained to participants in their local language (Runyankole) and informed written consent was obtained from each study participant who agreed to participate. Each participant was interviewed independently and the collected information was kept confidential. Study numbers were used instead of participants’ names to ensure confidentiality.

## Results

### Socio-demographic characteristics of participants

A total of 371 participants from the three sub-counties in Kiruhura district were recruited into the study. Forty percent of the participants were between ages 30 to 44 years (with median age of 40 and standard deviation 14.8 years) while 49% were males and 51% were females. Of these participants, 165 (44.5%) were from Kanyaryeru, 109 (26.1%) from Nyakashashara and 97 (29.4%) were from Sanga sub-counties. Majority of the participants were agro-pastoralists 181(49%), 165(45%) had attained primary education and 257(69%) were married.

### Communities’ awareness and perception about brucellosis

A majority of the participants (370, 99.3%) had ever heard about brucellosis which is commonly known as ‘brucella’ in the study areas. Of these, 339 (91.4%) had heard about brucellosis in their area of residence mainly from friends, (157, 42.4%) of the participants and 309(89.8%) said they heard about it in their area since the year 2000. Responses from the participants depicted that brucellosis affects all age groups and all sexes and 243(66.2%) mentioned that the disease is not seasonal. Additionally, those who had ever heard about brucellosis, 222(59.8%) had a household member who had ever suffered from the disease. Two thirds of the participants (251, 67.7%) mentioned that close proximity to wild animals is the major factor that contributes to the increase of brucellosis in the study areas (Table 1).

**Table 1:** Communities’ awareness and perception about brucellosis

**Variables**	**Frequencies (N = 371)**	**Percentages (%)**
**Ever heard about brucellosis**		
Yes	370	99.3
No	1	0.7
**Heard from whom?**		
Friend(s)	157	42.4
Health workers	73	19.7
Media	38	10.3
Patient of brucellosis	36	9.7
I/family member/people from the village	66	18.0
**Is brucella a health problem in this area?**		
Yes	339	91.4
No	32	8.6
**Since when?**		
Recent yrs (2000–2012)	309	89.8
I cannot remember	35	10.2
**Household member suffered from brucellosis**		
Yes	222	59.8
No	147	39.6
I don’t remember	2	0.6
**Most affected (sex)**		
Men	9	2.5
Women	38	10.4
Both sexes	311	84.7
I don’t know	9	2.5
**Is brucellosis seasonal?**		
Yes	69	18.8
No	243	66.2
I don’t know	55	15
**Factors for the increase**		
Close proximity to wild animals	251	67.7
Poverty	36	9.7
Unawareness about brucellosis	75	20.2
I do not know	9	2.4

### Communities’ knowledge about causes and symptoms of brucellosis

The majority of respondents (279, 75.2%) suggested joint and muscle pain as the major symptom of brucellosis in humans. A low proportion of strict pastoralists compared to the agro-pastoralist (38.0% vs 45.3%, *P* = 0.01) mentioned body weakness and weight loss (9.0% vs 13.8%, *P* = 0.02) respectively. Most participants (299, 81%) did not know about the symptoms of brucellosis in animals while only 52 (14%) believed that abortions in animals at six to seven months was a sign of brucellosis.

### Communities’ knowledge on symptoms and treatment of brucellosis

Up to 286 participants (77.1%) were aware that brucellosis presents like other illnesses and the most common illness mentioned was malaria (239, 64.4%). A higher proportion of participants (95.4%) knew that brucellosis in humans is treatable and the majority mentioned treatment with modern drugs (95.2%) and (84.1%) knew it is treatable in animals by seeking treatment from a veterinary officer (78.2%) see Table 2.

**Table 2:** Communities’ knowledge on symptoms and treatment of brucellosis

**Does it present like other illnesses?**	**Frequencies (N = 371)**	**Percentages (%)**
Yes	286	**77**.1
No	85	22.9
**Which other illnesses?**		
Malaria	239	64.4
Typhoid	28	7.5
Tuberculosis	19	5.1
I do not know	85	22.9
**Is brucellosis treatable in humans**		
Yes	354	95.4
No	5	1.4
I don’t know	12	3.2
**Kind of treatment sought for humans**		
Modern drugs	353	95.2
Traditional medicine	9	2.4
Both	7	1.9
I don’t know	2	0.5
**Is brucellosis treatable in animals**		
Yes	312	84.1
No	16	4.3
I don’t know	43	11.6
**Kind of treatment sought for animals**		
Veterinary officer	292	78.7
Treat them myself	73	19.7
I don’t know	6	1.6

### Communities’ knowledge about transmission of brucellosis in both humans and animals

The majority (360, 97.0%) of the participants from all occupations mentioned consumption of unpasteurized dairy products, especially milk, and eating of raw or half-cooked meat (333, 91.4%) as the most common mode of transmission from domestic animals to humans. Additionally, a high proportion (324, 87.3%) reported eating raw or half cooked meat as a mode of transmission of brucellosis from wild animals to humans. Eating contaminated pasture was reported as a common mode of transmission of brucellosis from domestic to domestic animals (212, 57.1%) and from wild animals to domestic animals (240, 64.7%). However, other transmission routes were less known such as; direct contact with birth products like placentas, inhalation of contaminated dust, sharing contaminated water sources, animals mating with infected animals and through artificial insemination if animals are not tested.

### Communities’ knowledge on prevention and risk factors of brucellosis

**Figure 1:**
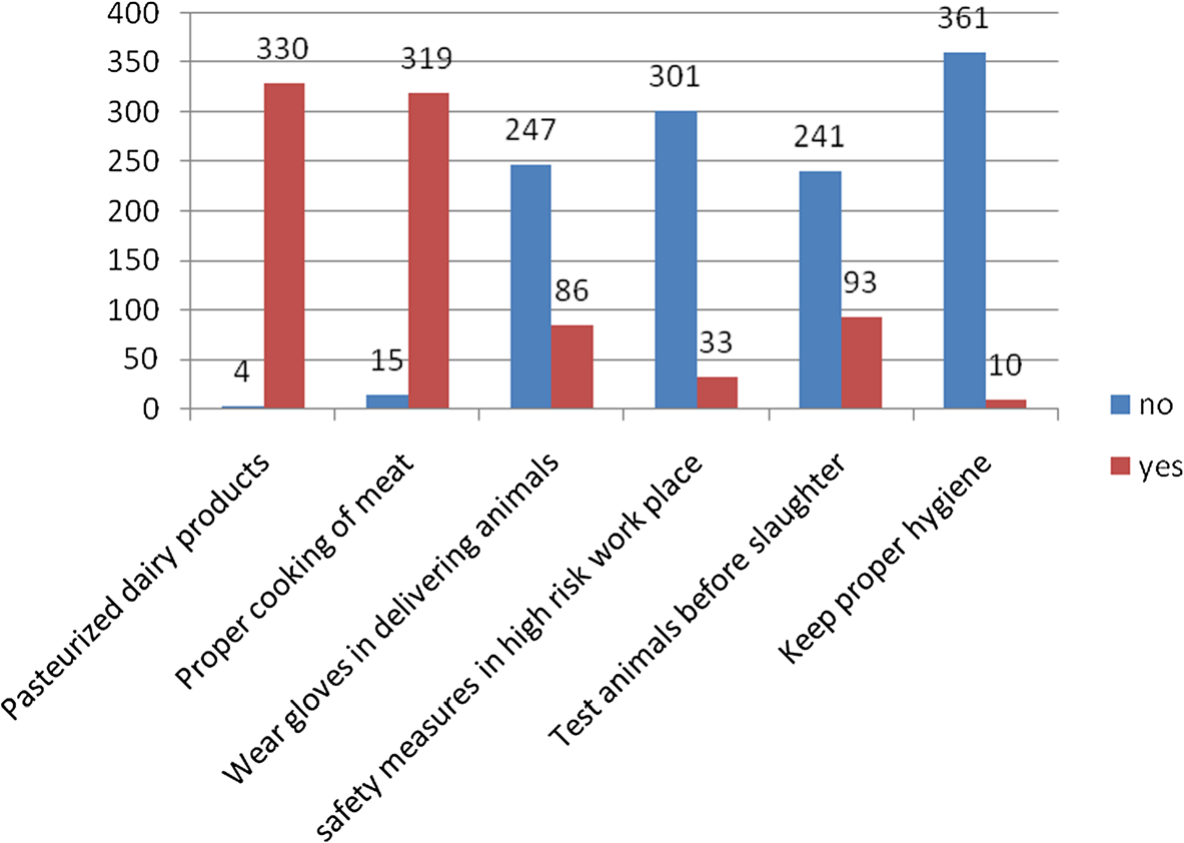
Communities’ knowledge on human prevention of brucellosis.

**Figure 2:**
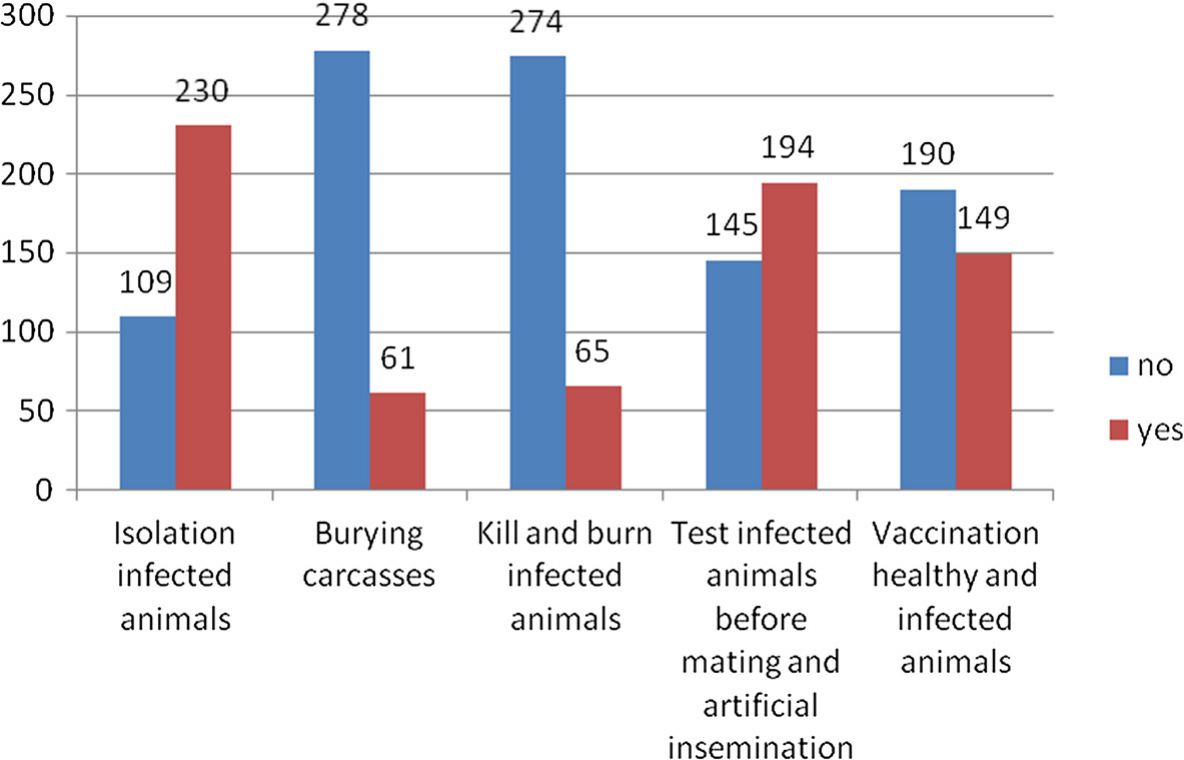
Communities’ knowledge on prevention of brucellosis in animals.

Majority of the participants (333, 89.8%) were aware that brucellosis is preventable in both humans and animals. However, only two methods of prevention of brucellosis were mainly mentioned: a high proportion (330, 98.8%) reported pasteurization of dairy products and 319 (95.5%) mentioned proper cooking of meat as the methods of prevention from animals to humans (Figure 1); while 230 (67.9%) mentioned isolation of infected animals from healthy ones (although majority said this is not usually practiced because of limited land) and 194 (57.2%) mentioned testing for infected animals before mating and artificial insemination as methods of prevention of brucellosis from animal to animal (Figure 2). Majority of the participants mentioned contaminated dairy products (340, 91.6%) and direct contact with contaminated birth products (212, 57.1%) as the most common risk factors of brucellosis.

### Association of participants’ socio-demographic characteristics with participants’ knowledge on symptoms, transmission, prevention and treatment of human and animal brucellosis

Knowledge of symptoms of brucellosis in humans was associated with agro-pastoralists (aOR: 2.53, CI: 1.28-5.00). High knowledge of preventive methods in humans (aOR: 2.79, CI: 1.02-3.31) and modern drugs as a choice of effective treatment (aOR: 3.69, CI: 1.01-13.36) were associated with females. High knowledge of preventive methods in humans was also associated with participants’ who had attained primary education (aOR: 2.48, CI: 1.21-5.49) as well as agro-pastoralists (aOR: 1.76, CI: 0.09-3.09). Low knowledge of symptoms in animals among all study participants was also associated with the age group 30–44 and 45–59 years (aOR: 3.48, CI: 1.25-9.70) and (aOR: 4.15, CI: 1.31-13.13) respectively.

### Association of participants’ socio-demographic characteristics with participants overall knowledge

Overall knowledge was categorized using the 50^th^ percentile as cut off and participants who had moderate knowledge were 197 (53.1%), and those with high knowledge 75th percentile were 174 (46.9%). Factors significantly associated with overall knowledge were agro-pastoralists (aOR: 2.08, CI: 1.17-3.71) compared to pure pastoralists and those who reported that the disease was a health problem (aOR: 0.18, CI: 0.06-0.56) compared to those who said it was not (Table 3).

**Table 3:** Association of participants’ socio-demographic characteristics with participants overall knowledge

**Variables**	**Level of knowledge, n (%)**		**Unadjusted OR (95% CI)**	**Adjusted OR (95% CI)**
	**Moderate 197 (53.1%)**	**High 174 (46.9%)**		
**Sub–county**				
Kanyaryeru	89(53.9)	76(46.1))	1	
Sanga	50(51.6)	47(48.5.0)	1.10(0.67–1.85)	1.29(0.73–2.28)
Nyakashashara	58(53.2)	51(46.8)	1.02(0.63–1.67)	1.08(0.64–1.84)
**Sex**				
Male	92(50.3)	91(49.7)		
Female	105(55.8)	83(44.2)	0.80(0.53–1.20)	0.87(0.55–1.36)
**Age**				
18–29	44(62.7)	26(37.1)	1	
30–44	77(51.7)	72(48.3)	1.58(0.88–2.83)	1.65 (0.88–3.12)
45–59	45(47.4)	50(52.6)	1.88(1.00–3.53)	1.90(0.93–3.84)
60**+**	31(54.4)	26(45.6)	1.42(0.70–2.89)	1.54(0.67–3.53)
**Education**				
No formal education	75(59.1)	52(40.9)	1	
Primary	82(49.7)	83(50.3)	1.46(0.92–2.33)	1.47(0.72–2.49)
Secondary	36(48.7)	38(51.3)	1.52(0.85–2.71)	1.53(0.65–3.09)
Tertiary	4(80.0)	1(20.0)	0.36(0.04–3.32)	0.32(0.03–3.50)
**Occupation**				
Farmer	57(63.3)	33(36.7)	1	
Pastoralist	63(63.0)	37(37.0)	1.01(0.56–1.83)	1.08(0.59–2.26)
Agro–pastoralists	77(42.5)	104(57.5)	2.33(1.39–3.92)	2.08(1.02–3.27)*
**Is brucellosis a health problem?**				
Yes	170(50.2)	169(49.8)	1	
No	27(84.4)	5(15.6)	0.19(0.07–0.50)	0.18(0.06–0.56)
**Is brucellosis seasonal?**				
Yes	36(52.2)	33(47.8)	1	
No	123(50.6)	20(49.4)	1.06(0.62–1.82)	0.85(0.47–1.54)
Do not know	35(63.6)	20(36.4)	0.62(0.30–1.29)	0.48(0.22–1.04)

*variables *P*-value < = 0.05 (* = 0.03). Level of knowledge was obtained using the 50^th^ and 75^th^ percentile. Overall knowledge was coded as 0 for moderate knowledge and 1 for high knowledge.

## Discussion

Knowledge and perceptions about brucellosis among high risk groups are crucial in influencing the health seeking behavior of patients as well as controlling its transmission in animals and humans in communities. Our study, that aimed at assessing these aspects on brucellosis, found that 197 (53.1%) had moderate overall knowledge on brucellosis symptoms, transmission, treatment, prevention and risk factors among the participants. A majority of the participants (99.3%) in our study had ever heard about brucellosis (commonly known as ‘brucella’). The main source of information about the disease was from friends (42.4%). Contrary to this finding, a study in central Asia [19] found that 31% of the respondents had heard about brucellosis from human doctors. The different sources of information highlight a need for multiple communication channels to transmit and improve public knowledge about brucellosis through the media, social gatherings and schools as well as ensuring that knowledge being transmitted is standardized through frequent follow-up with health education talks on biomedical knowledge of the disease from health and veterinary.

In regard to perceptions about brucellosis, although most of the participants (311, 84.7%) believed that the disease does not segregate between age groups and sex, (243, 66.2%) believed it is not seasonal but occurs throughout the year. two thirds of the participants (251, 67.7%) mentioned that close proximity to wild animals is the major factor that contributes to the increase of brucellosis in the study areas and the majority of the participants (89.8%) believed that brucellosis became a health problem from around the year 2000 when wildlife–human–domestic animal interaction increased as a result of drought in the area. This increasing frequency and severity of drought, particularly in the semi-arid areas of the cattle corridor has resulted in a lack of water and natural pasture [20]. From our study area, the Lake Mburo National Park Authority in 2009 reported that farmers, mostly from Sanga, Kanyaryeru, and Nyakashashara sub-counties, moved over 15,000 cows to the park that competed with wild animals for food and water and increased the risk for the transmission of animal diseases [21]. Wild animals such as: buffaloes, impalas and zebras are common in grazing areas, mixing freely with livestock. Brucellosis prevalence in buffaloes has been reported from Egypt (10.0%) and Pakistan (5.05%) [22]. With this increasing interaction that poses a threat to both humans, domestic animals and wild animals, there is need for collaborative efforts from the health and veterinary sectors, the Uganda Wildlife Authority as well as the communities in order to control brucellosis by implementing restrictions on animal movements.

All participants had a high level of knowledge of the clinical signs of brucellosis in humans, mostly recurrent fever and joint and muscle pains (Table 3). This finding is similar to that seen in a study in Central Asia [19] where 84% of owners of small ruminants mentioned joint ache and other limb problems; and half of them mentioned fever as a symptom of brucellosis in humans. Our findings may be due to the fact that the study area is an endemic setting, thus the participants have basic knowledge of the disease. Contrary to this, a study done among herdsmen in Ghana [23] found that only 4.5% knew at least one symptom of brucellosis in humans. A majority (77%) of the participants in our study were aware that brucellosis presents like other common illnesses and 64% reported that its symptoms were similar to those of malaria. This is crucial because in humans, brucellosis is often easily misdiagnosed as other febrile syndromes such as malaria and typhoid fever, thereby resulting in underreporting and hence misdirected treatments [3].The ability of our respondents to identify symptoms of brucellosis in humans as well as other febrile illnesses is crucial in seeking health care and minimizes misdiagnosis of the disease as well as unwarranted treatment. With this increased knowledge, there is need to increase the effective demand for services by highlighting prompt treatment for brucellosis as well as the provision of equipped health facilities to address the demand.

Conversely, very low knowledge of the symptoms of brucellosis in animals was depicted by the respondents. Only 70 (19%) knew the symptoms of brucellosis in animals. A small proportion (14%) mentioned that abortion in animals was a major sign of the disease, findings similar to studies done in cattle keeping communities of Nigeria ([24]) and Central Asia [19], where abortion was mentioned by 11% of the participants. This low knowledge may be due to the different perceptions that pastoralists have concerning infertility, reduced milk production and abortions since perception of a risk is influenced by such factors as life experience and culture [25] as well as inadequate knowledge of the disease in animals. Contrary to the above, a study done in Egypt [18] found high knowledge (94.4%) of the clinical signs (abortions and low milk production) in animals and concluded that this finding was consistent with the endemic situation in Egypt. Since low knowledge of brucellosis in animals poses a zoonotic threat to public health, it is important to provide health education on animal brucellosis since the source of human infection resides in domestic or wild animal reservoir and therefore, prevention of human brucellosis depends predominantly on the control of the disease in animals [26].

Participants were knowledgeable on transmission routes of brucellosis which were; consumption of unpasteurized dairy products (97%) and eating of raw or half-cooked meat in humans (91.4%) while in animals eating contaminated pasture (97.4%). Additionally, most participants (87.3%) were aware that eating game meat was a source of transmission from wild animals to humans. The increasing wildlife–human–domestic animal interface, including the consumption of game meat around the world has recently attracted concerns and is challenging [27] since 75% of emerging infectious diseases are zoonoses that are predominantly associated with wildlife [28] which clearly highlights an increasing threat arising from these animal species. However, other animal to human and within animal transmission routes such as direct contact with infected birth products or aborted materials like placentas [29], and inhalation of contaminated dust, animals mating with infected animals and through artificial insemination [30] were less known by the study participants. Similar studies elsewhere have shown less community knowledge and understanding regarding such hazards as infected placental materials and contaminated products, as was seen in Central Asia [19] and in Ghana [23], where only 12.9% of the respondents knew the transmission from cattle to humans. There is a need for increased public health education and behavioral change communication with emphasis on various modes of transmission from animal to animal (both domestic and wild animals) and from animal to humans in order to better control the disease in endemic areas.

Almost all the participants were knowledgeable about treatment of brucellosis in both humans and animals. They reported use of modern drugs in humans (95%) and seeking veterinary care for animals (84%) as the best options. We view this as a good indicator that would influence better health care seeking behavior and uptake of public health messages. Furthermore, most of the participants (89.8%) were aware that brucellosis is preventable in both humans and animals. However, only two methods of prevention from animals to humans and within animals were commonly mentioned: pasteurization of dairy products (88.9%) and proper cooking of meat (86.0%) to prevent transmission to humans; and isolation of infected animals (62.0%) from healthy ones as well as testing animals before mating and artificial insemination (52.3%). Ironically, it was noted that majority of the participants did not practically isolate the sick animals because of lack of facilities for isolation of suspected and/or infected animals, yet this is one of the major risk factors for disease transmission between animals as susceptible animals can be infected via contact with sick animals or contact with aborted materials or products of parturition [29].

Although participants were aware of the importance of testing animals for brucellosis before mating and vaccination of healthy animals, they cited high costs as a hindrance. This is compounded by the fact that there is no vaccine that has been proven to be safe and to provide significant degree of protection in wild animals species [31], hence a risk remains for spill-over from wild to domestic animals as a result of interaction in marginal grazing areas. A previous study investigating patient perceptions of brucellosis in Greece [32] found that around 44% of farmers would not allow veterinary investigation for fear of undesirable effects on their herds. This indicates that underreporting is likely to be a problem hindering brucellosis control in the communities. This difference between knowledge and actual practice indicates that high knowledge of a disease does not necessarily go hand in hand with accurate behavior and practices, as other factors may come into play. Therefore, there is need for continuous innovative preventive and control strategies such as laboratory-backed surveillance, equipped laboratories, training, education and communication on brucellosis in the communities in order to reduce transmission.

In our multivariate logistic regression analysis, agro-pastoralism as an occupation was a predictor of high overall knowledge of brucellosis. Studies in similar settings of pastoralist [33,34] and agro-pastoral communities [35] in Tanzania found that agro-pastoralism as an occupation was predictive of high biomedical overall knowledge of pulmonary tuberculosis (PTB). The similarity in our study maybe as a result of sedentarization of pastoralists in Uganda’s cattle corridor [36], where this change of lifestyle may have brought about to improve access to health and social services [37] as well as veterinary extension services. The cattle corridor occupies a significant proportion of approximately 44% of Uganda’s total land area. It stretches from the south through the districts of Ankole and northern parts of Buganda to the north central part of Uganda [38]. This area is semi-arid, and has suitable climatic conditions that make it conducive to cattle rearing.

Mobility in pastoralist communities has been cited as a great hindrance to access to knowledge as well as health care and veterinary extension services because of the geographical, social and cultural environment [39]. This may be curbed by bringing health and veterinary services such as mobile clinics and social services closer to the people who do not easily access them.

Participants who knew that brucellosis was a health problem in the area was significantly associated with overall knowledge of brucellosis.There is need for more health education on brucellosis for better prevention and control of the disease in the communities.

At sub scale analysis for each domain of knowledge, high knowledge of choice of effective treatment as modern drugs was associated with being female. Conversely, contrary findings were found in a study in pastoral communities in Ethiopia which found an association between males and high knowledge of choice of treatment with modern tuberculosis drugs [34]. Findings in our study may be as a result of differences in health seeking behaviour between females and males and/or economic independence between the genders in these communities. However, low knowledge of symptoms in animals among all study participants was also associated with the age group 30–59 years although this was not significant when analyzing overall knowledge. This result may be due to different perceptions on symptoms of the disease in terms of age and experience. Therefore, health education on brucellosis targeting age groups and both sexes is crucial in order to change the perceptions of the people to more biomedical knowledge for better management and control of the disease.

A possible limitation to this study is that the selection of participants was based on systematic sampling which may have brought in errors and biases. However, we believe that this was controlled for in the selection process since households were homogenous in nature. The second limitation was during data collection where some respondents (household heads) were away with their herds in search of water and pasture since it was a dry season. This was minimized by making appointments and for those who were not available; their spouses were interviewed after consenting to the study. We recommend a future study to explore ways of promoting health education on brucellosis in the communities as a control strategy of the disease.

## Conclusion

Knowledge on three transmission routes of brucellosis in humans and animals was nearly universal. Participants were more knowledgeable about brucellosis symptoms in humans than in animals. Participants had moderate overall knowledge of brucellosis in both humans and animals. Majority believed the disease affects both sexes and nearly three thirds of the respondents believed that close proximity to wildlife contributes to the presence of the disease in the area. This highlights the need for collaboration between the public health, veterinary sectors and wildlife authorities in the provision of health education and information on the cause, symptoms, transmission and prevention of brucellosis for better management of the disease in the communities.

## Competing interests

The authors declare that they have no competing interests.

## Authors’ contributions

Conception of the study (CK, SM, IBR, BBA, ER): study design (CK, AM, SM, BBA, IBR, SNK, ER); data collection (CK, BBA, SM); data analysis (CK, FM and JS), drafting manuscript (CK, BBA, ER), critical revision of manuscript (AM, FM, SM, IBR, JS, SNK, BBA, ER), over all supervision of the work (SM, AM, ER). All authors read and approved the final version of the manuscript.

## Pre-publication history

The pre-publication history for this paper can be accessed here:

http://www.biomedcentral.com/1471-2458/14/242/prepub

## References

[B1] Ariza J Bosilkovski M Cascio A Colmenero JD Corbel MJ Falagas ME Memish ZA Roushan MR Rubinstein E Sipsas NV Solera J Young EJ Pappas G Prospectives for the Treatment of Brucellosis in the 21st Century: the Ioannina Recommendations PLoS Med 2007 4 e317 10.1371/journal.pmed.0040317 18162038 PMC2222927

[B2] Young EJ Mandell GL, Bennet JE, Dolin R Brucella species Principles and Practice of Infectious Diseases 2000 5 Philadelphia: Churchill-Livingstone 2386 2393

[B3] Bax HI Van Veelen ML Gyssen IC ‘Brucellosis, an uncommon and frequently delayed diagnosis’. [Links] Neth J Med 2007 2 352 355 17954956

[B4] John K Fitzpatrick J French N Kazwala R Kambarage D Mfinanga SG MacMillan A Cleaveland S Quantifying risk factors for human brucellosis in Rural Northern Tanzania PLoS ONE 2010 5 4 10.1371/journal.pone.0009968 PMC2848606 20376363

[B5] WHO Brucellosis in Humans and Animals (World Health Organisation) 2006 http://www.who.int/csr/resources/…/Brucellosis.pdf

[B6] Corbel M Brucellosis in Humans and Animals: FAO, OIE, WHO Available: http://www.who.int/csr/resources/publications/Brucellosis.pdf. Accessed 2012 May 7. 2006

[B7] Smits HL Cutler SJ Contributions of biotechnology to the control and prevention of brucelliosis in Africa Afr J Biotechnol 2004 12 3 631 636

[B8] Roth F Zinsstag J Orkhon D Chimid-Ochir G Hutton G Cosivi O Carrin G Otte J Human health benefits from livestock vaccination for brucellosis: case study Bull World Health Organ 2003 81 867 876 14997239 PMC2572379

[B9] Godfroid J Cloeckaert A Liautard JP Kohler S Fretin D Walravens K Garin-Bastuji B Letesson JJ From the discovery of the Malta fever’s agent to the discovery of a marine mammal reservoir, brucellosis has continuously been a re-emerging zoonosis Vet Res 2005 36 313 326 10.1051/vetres:2005003 15845228

[B10] Galukande M Muwazi S Mugisa DB Aetiology of low back pain in Mulago Hospital, Uganda Afr Health Sci 2005 5 164 167 [PMC free article] [PubMed]) 16006225 PMC1831909

[B11] Makita K Fevre EM Waiswa C Kaboyo W De Clare Bronsvoort BM Eisler MC Welburn SC Human brucellosis in urban and peri-urban areas of Kampala, Uganda Ann N Y Acad Sci 2008 1149 309 311 10.1196/annals.1428.015 19120236

[B12] Bernard F Vincent C Matthieu L David R James D Tuberculosis and brucellosis prevalence survey on dairy cattle in Mbarara milk basin (Uganda) Prev Vet Med 2005 67 4 267 281 10.1016/j.prevetmed.2004.11.002 15748756

[B13] Magona JW Walubengo J Galiwango T Etoori A Seroprevalence and potential risk of bovine brucellosis in zerograzing and pastoral dairy systems in Uganda Trop Anim Health Prod 2009 41 8 1765 1771 10.1007/s11250-009-9375-y 19468854

[B14] National Adaptation Programmes of Action on Climate Change for Uganda (NAPA) Uganda National Adaptation Programmes of Action 2007 Kampala, Uganda

[B15] Rhyan JC Brown K, Bolin C Brucellosis in terrestrial wildlife and marine mammals Emerging Diseases of Animals 2000 Washington

[B16] Uganda Bureau of Statistics Housing Census 2002 http://www.ubos.org/onlinefiles/uploads/ubos/pdf%20documents/2002%20CensusPopnSizeGrowthAnalyticalReport.pdf

[B17] Haydon DT Cleaveland S Taylor LH Laurenson MK Identifying reservoirs of infection: a conceptual and practical challenge Emerg Infect Dis 2002 8 12 1468 1473 12498665 10.3201/eid0812.010317 PMC2738515

[B18] Sirmatel F Turker M Bozkurt AI Evaluation of the methods used for the serologic diagnosis of brucellosis Mikrobiyol Bul 2002 36 161 167 12652868

[B19] Grahn C Brucellosis in Small Ruminants-an Investigation of Knowledge, Attitudes and Practices in Peri-Urban Farming around the Region of Dushanbe, Tajikistan 2013 Uppsala 1652 8697 http://epsilon.slu.se. 38. 2013

[B20] The National Policy for Disaster Preparedness and Management The National Policy for Disaster Preparedness and Management 2010 http://www.opm.go.ug/assets/media/resources/8/Disaster%20Policy.pdf

[B21] World Bank Ministry of Foreign Affairs. Uganda Dairy Supply Chain Risk Assessment: February 2011 2011 http://www.agriskmanagementforum.org/sites/agriskmanagementforum.org/files/Documents/UgandaDairy10.pdf)

[B22] Gul ST Khan A The Epidemiology and Epizootology of Brucellosis, Volume 27(3) A Review Pakistan Vet J 2007 Faisalabad, Pakistan: Department of Veterionary Pathology, University of Agriculture 145 151

[B23] Kennedy Kwasi A Gloria Ivy M Naomi N George Kwasi N David M Kwame George A Smits HL Knowledge, Attitudes and Practices (KAP) of Herdsmen in Ghana with respect to Milk-Borne Zoonotic Diseases and the Safe Handling of Milk J Basic Appl Sci Res 2011 1 10 1556 1562

[B24] Hezekiah K AdesokanI P AlabiI Judy A StackII S CadmusI B Knowledge and practices related to bovine brucellosis transmission amongst livestock workers in Yewa, south-western Nigeria J S Afr Vet Assoc 2013 84 1 Pretoria 2013 10.4102/jsava.v84i1.121 23718254

[B25] Food and Agriculture Organization Brucella Melitensis in Eurasia and the Middle East. FAO Animal Production and Health Proceedings. No 10. Rome 2010

[B26] Thakur SD Kumar R Thapliyal DC Human brucellosis: review of an under-diagnosed animal transmitted diseases J Commun Dis 2002 34 287 301 14710861

[B27] Cima G Wildlife, Trade, Susceptibility Amplify Food Risks JAVMA 2012 Available from: https://www.avma.org/News/JAVMANews/Pages/120215a.aspx [cited 1 February 2012] [PubMed] 10.2460/javma.240.4.352 22309005

[B28] Smith KM Anthony SJ Switzer WM Epstein JH Seimon T Jia H Sanchez MD Zoonotic viruses associated with illegally imported wildlife products PLoS One 2012 7 e29505 [PMC free article] [PubMed] 10.1371/journal.pone.0029505 22253731 PMC3254615

[B29] Corbel M Brucellosis. In Fertility and Infertility in Veterinary Practice. 4th edition. Edited by Laing J. Bailliere Tindall: ELBS; 1988:190–221 cited in Hannah R Holt, Mahmoud M Eltholth Yamen M Hegazy, Wael F El-Tras, Ahmed A Tayel and Javier Guitian. Brucella spp. infection in large ruminants in an endemic area of Egypt: cross-sectional study investigating seroprevalence, risk factors and livestock owner's knowledge, attitudes and practices (KAPs) BMC Public Health 2011 11 341 doi:10.1186/1471-2458-11-341. http://www.biomedcentral.com/1471-2458/11/341# 10.1186/1471-2458-11-341 21595871 PMC3121632

[B30] Memish ZA Almuneef M Mah MW Qassem LA Osoba AO Comparison of the Brucella Standard Agglutination Test with the ELISA IgG and IgM in patients with Brucella bacteremia Diagn Microbiol Infect Dis 2002 44 129 132 10.1016/S0732-8893(02)00426-1 12458117

[B31] Godfroid J Brucellosis in wildlife Rev Sci Tech Off Int Epizoot 2002 21 277 286 10.20506/rst.21.2.1333 11974615

[B32] Karimi A Alborzi A Rasooli M Kadivar MR Nateghian AR Prevalence of antibody to Brucella species in butchers, slaughterers and others East Mediterr Health J 2003 9 178 184 15562749

[B33] Gele AA Bjune G Abebe F Pastoralism and delay in diagnosis of TB in Ethiopia BMC Public Health 2009 9 5 14 10.1186/1471-2458-9-5 19128498 PMC2628652

[B34] Mengistu L Gobena A Gezahegne M Girmay M Dawit S Gunnar B Fekadu A Knowledge and perception of pulmonary tuberculosis in pastoral communities in the middle and lower Awash Valley of Afar region, Ethiopia BMC Public Health 2010 10 187 http://www.biomedcentral.com/1471-2458/10/187 10.1186/1471-2458-10-187 20380747 PMC2867998

[B35] Al-Tawfiq JA AbuKhamsin A 24-year study of the epidemiology of human brucellosis in a health-care system in Eastern Saudi Arabia J Infect Public Health 2009 2 81 85 10.1016/j.jiph.2009.03.003 20701865

[B36] United Nations Development Programme Between a Rock and Hard Place: Armed Violence in African Pastoral Communities. UNDP report 2007 http://www.eldis.org/go/home&id=45309&type=Document#.UyGprfsrmFc

[B37] Swai ESSL Human brucellosis: seroprevalence and risk factors related to high risk occupational groups in Tanga Municipality, Tanzania Zoonoses Pub Health 2009 56 183 187 10.1111/j.1863-2378.2008.01175.x 18811674

[B38] Government of Uganda Draft Rangeland Management Policy of Uganda 2011

[B39] Queipo-Ortuño MI Colmenero JD Reguera JM García-Ordoñez MA Pachón ME Gonzalez M Morata P Rapid diagnosis of human brucellosis by SYBR Green I-based realtime PCR assay and melting curve analysis in serum samples Clin Microbiol Infect 2005 11 713 718 10.1111/j.1469-0691.2005.01202.x 16104985

